# In vitro susceptibility of *Borrelia burgdorferi* isolates to three antibiotics commonly used for treating equine Lyme disease

**DOI:** 10.1186/s12917-017-1212-3

**Published:** 2017-09-29

**Authors:** Sanjie Caol, Thomas Divers, Mark Crisman, Yung-Fu Chang

**Affiliations:** 1 Department of Population Medicine and Diagnostic Sciences, College of Veterinary Medicine, Cornell University, Ithaca, NY 14853 USA; 2 Currently Research Center of Swine Disease, College of Veterinary Medicine, Sichuan Agricultural University, Chengdu, 611130 China; 3 Department of Clinical Sciences College of Veterinary Medicine, Cornell University, Ithaca, NY 14853 USA; 4 Virginia-Maryland College of Veterinary Medicine, Blacksburg, VA 24061 USA

**Keywords:** Horse, *Borrelia burgdorferi*, Lyme, Ceftiofur, Metronidazole, Doxycycline

## Abstract

**Background:**

Lyme disease in humans is predominantly treated with tetracycline, macrolides or beta lactam antibiotics that have low minimum inhibitory concentrations (MIC) against *Borrelia burgdorferi.* Horses with Lyme disease may require long-term treatment making frequent intravenous or intramuscular treatment difficult and when administered orally those drugs may have either a high incidence of side effects or have poor bioavailability. The aim of the present study was to determine the in vitro susceptibility of three *B. burgdorferi* isolates to three antibiotics of different classes that are commonly used in practice for treating *Borrelia* infections in horses.

**Results:**

Broth microdilution assays were used to determine minimum inhibitory concentration of three antibiotics (ceftiofur sodium, minocycline and metronidazole), for three *Borrelia burgdorferi* isolates. Barbour-Stoner-Kelly (BSK K + R) medium with a final inoculum of 10^6^ *Borrelia* cells/mL and incubation periods of 72 h were used in the determination of MICs. Observed MICs indicated that all isolates had similar susceptibility to each drug but susceptibility to the tested antimicrobial agents varied; ceftiofur sodium (MIC = 0.08 μg/ml), minocycline hydrochloride (MIC = 0.8 μg/ml) and metronidazole (MIC = 50 μg/ml).

**Conclusions:**

The MIC against *B. burgorferi* varied among the three antibiotics with ceftiofur having the lowest MIC and metronidazole the highest MIC. The MIC values observed for ceftiofur in the study fall within the range of reported serum and tissue concentrations for the drug metabolite following ceftiofur sodium administration as crystalline-free acid. Minocycline and metronidazole treatments, as currently used in equine practice, could fall short of attaining MIC concentrations for *B. burgdorferi*.

## Background

Lyme borreliosis, the most common tick-transmitted disease in the northern hemisphere, is caused by Gram-negative spirochetes of the *Borrelia burgdorferi* sensu *lato* complex [1]. In North America, *B. burgdorferi* sensu stricto is the cause of Lyme disease although several strains of that organism are found [2]. Adult horses in some endemic areas of the U.S. have seroprevalence rates of 33% or greater indicating a high incidence of *B. burgdorferi* exposure [3–7]. Lyme disease in horses is documented by numerous case reports [8–15], but the proportion of seropositive horses with clinical Lyme disease is unknown. Lyme disease in humans is treated predominantly with tetracyclines or beta lactam antimicrobials with low minimum inhibitory concentrations (MIC) against *Borrelia burgdorferi* [16]. Successful treatment of chronic infections is believed to require longer treatment duration than for early infections [17]. Early infections are rarely recognized in the horse due to absence of erythema migrans. Antibiotic treatment, mostly minocycline or doxycycline given per os, for four or more weeks’ duration is common in seropositive horses with signs thought to be associated with Lyme disease [18].

As with most other bacterial diseases, susceptibility of the causative agent to the antibiotics used for therapy is an important prerequisite for antibiotic selection and successful treatment. Multiple in vitro studies have indicated susceptibility of *B. burgdorferi* to several antibiotics including amoxicillin, azithromycin, several cephalosporins such as ceftriaxone and cefuroxime, and most of the tetracycline group of antibiotics [19–28]. Resistance has been demonstrated to trimethoprim sulfas and flouroquinolones, two commonly used oral antibiotics in horses [25, 29]. Differences in the antibiotic susceptibilities of different strains and forms (motile or cysts) of *B. burgdorferi* sensu stricto have been reported [25, 30, 31]. Most in vitro testing is performed on viable, motile *B. burgdorferi,* as they are readily available for testing and assumed to be the *Borrelia* stage most commonly causing disease [1]. A persistent (cystic, non-growing) form of *Borrelia* has been associated with, but is not proven to cause, chronic Lyme disease. This form of *Borrelia* is known to have a very high resistance pattern to the antibiotics commonly used for acute infections but has a moderate sensitivity to metronidazole [30–32].

In adult horses, use of many of the antibiotics recommended for treatment of Lyme disease in humans (tetracyclines, beta lactams and macrolides) is associated with a high incidence of diarrhea (beta lactams and macrolides) when administered orally and all three classes of antibiotics have highly variable and frequently low oral bioavailability in the horse [33–38]. In addition, parenteral administration of tetracycline or beta lactam drugs that are known to be effective in vitro against *Borrelia spp.* can cause injection site reactions following repeated intramuscular administration. Prolonged intravenous administration of tetracycline can cause renal failure and thrombophlebitis [39].

The aim of the present study was to determine the in vitro susceptibility of three *B. burgdorferi* isolates to three antibiotics that might be of practical use for treating *Borrelia* infections in horses.

## Methods

The susceptibility of clinical isolates of *B. burgdorferi* to three different classes of antibiotics was evaluated by measurement of their minimum inhibitory concentrations (MICs). The three antibiotics evaluated in the study were antibiotics commonly used in equine practice and administered either orally (minocycline and metronidazole) or intramuscularly (ceftiofur) in treating equine Lyme disease.

### *Borrelia burgdorferi* isolates

Three *B. burgdorferi* sensu stricto isolates obtained from the collection of Dr. Yung-Fu Chang at the Department of Population Medicine and Diagnostic Sciences, College of Veterinary Medicine, Cornell University were tested for antibiotic susceptibility. Isolates were obtained from skin biopsies of ponies that had been experimentally infected by attaching adult ticks (*Ixodes scapularis*), collected in a forested area of Westchester County, New York.

### Antimicrobial agents

The antibiotics tested were ceftiofur sodium (Sigma-Aldrich, USA), minocycline hydrochloride (MP Biomedicals, LLC), and metronidazole (Sigma-Aldrich, USA). All antibiotics were obtained as United States Pharmacopeia reference standard powders and were prepared according to the manufacturer’s recommendations. The concentration ranges used for testing were: ceftiofur sodium 0.04–2.56 μg/ml; minocycline hydrochloride 0.20–12.80 μg/ml; and metronidazole 12.50–800.00 μg/ml. The ranges were chosen according to data in the literature [24, 30].

### Culturing *B. burgdorferi*

*Borrelia burgdorferi* was cultured in Barbour-Stoner-Kelly (BSK K + R) medium [40]. The cultures were incubated at 34 °C with 5% CO_2_ and maintained in sterile 15 mL Corning tubes (cat.# 89,093–186 from VWR, Radnor, PA USA) with 10 mL BSK K + R medium for about 3–5 days.

### In vitro testing of the antibacterial agents

Broth microdilution procedures were used in the determination of minimum inhibitory concentrations (MICs) [28, 40, 42]. For this procedure, MICs were determined using sterile 48-wells Tissue Culture Plates (VWR International, LLC, USA). The last column served as the negative control and contained BSK K + R medium only. In the wells of all other columns, 900 μL of *B. burgdorferi* culture was added at a final density of 10^6^ cells/mL, determined in a Neubauer counting chamber (Brand, Wertheim, Germany). The second column, which served as the positive control, contained no antimicrobial agent. From the third column onward, 100 μL portions of two fold decreasing concentrations of antibiotics were added. Each antimicrobial agent was tested in triplicate assays, to demonstrate reproducibility of the assay, before being sealed with adhesive plastic and incubated at 34 °C in aerobic conditions for 72 h [28, 30]. Following incubation, all wells were examined by the same observer (SC) for visible growth and motility of isolates by dark-field microscopy. The lowest antibiotic concentration at which isolates demonstrated no visible growth and motility was determined for each antibiotic [30]. The MIC was determined three times for each isolate and provided as descriptive data in Table 1. The final MIC was defined as the lowest concentration of an antimicrobial that inhibits the visible growth and motility of a microorganism after 72 h of incubation [30] and reported as the mode value for all isolates (Table 1).

**Table 1:** Minimum inhibitory concentrations (MICs) of three antibiotics for individual *Borrelia burgdorferi* isolates

Isolate		MIC^a^ (μg/ml)		
		Ceftiofur	Minocycline	Metronidazole
	Experiment	MIC	MIC	MIC
6342	1	0.08	0.8	25
	2	0.08	0.4	50
	3	0.04	0.8	50
TLSH-916	1	0.08	0.8	50
	2	0.04	0.8	25
	3	0.08	0.4	25
21,343/3	1	0.04	0.8	50
	2	0.08	0.8	50
	3	0.08	0.4	25
Mode Value		0.08	0.8	50

^a^MIC_,_ are reported for individual isolates and assays [1–3]; the mode value is reported as the final MIC

## Results

The in vitro susceptibility of three isolates of *B. burgdorferi* to three antibiotics was tested. All of the isolates were susceptible to all tested antibiotics at some concentration. Table 1 shows the MICs values of each antibiotic tested against the individual *B. burgdorferi* isolates. According to MIC breakpoints^a^ (Table 1), the lowest drug concentrations to inhibit all the strains was 0.08 μg/ml for ceftiofur sodium, 0.80 μg/ml for minocycline hydrochloride, and 50 μg/ml for metronidazole. Complete inhibition of motility was noted at the MIC concentration for each antibiotic. Ultimately, the *B. burgdorferi* strains tested were most susceptible in vitro to ceftiofur and least susceptible to metronidazole.

## Discussion

There are multiple reports on in vitro susceptibility of isolates of *B. burgdorferi* to macrolides, tetracyclines and beta lactam antibiotics [19–28]. However, susceptibility to ceftiofur sodium has not been previously reported and susceptibility to metronidazole has only been reported twice with conflicting findings [27, 31]. Ceftiofur sodium is a third-generation cephalosporin approved for use in horses as both a sodium salt formulation with a rapid absorption and elimination time, and in a crystalline formulation intended for IM administration with a slowed absorption and greater area under the curve (AUC). Ceftiofur is rapidly metabolized to desfuroylceftiofur in the horse but the in vitro activity of desfuroylceftiofur is almost identical to that of ceftiofur for Gram-negative bacteria and similar for most Gram-positive bacteria tested [43]. Desfuroylceftiofur was not available for testing in this study and ceftiofur was used instead. In vitro activity testing against *B. burgdorferi* with either desfuroylceftiofur or ceftiofur has not previously been reported. In vitro testing of ceftiofur against *B. burgdorferi* seemed pertinent because cephalosporins have variable in vitro activity against *B. burgdorferi* with ceftriaxone, cefotaxime, cefdinir, and cefixime having a high activity while others such as cefetamet-pivoxil, ceftibuten, and cefpodoxime-proxetil were ineffective in vitro [22]. We were particularly interested in determining the MIC of ceftiofur because the crystalline form of the drug is approved to be administered IM in horses on days one, four and then weekly, and will maintain serum ceftiofur and desfuroylceftiofur combined concentrations >0.22 μg/ml throughout the treatment period and for at least six days following the end of treatment [44–46]. This serum concentration would be well above the MIC of ceftiofur against *B. burgdorferi* reported here. The most important determinant of efficacy with a time dependent antimicrobial such as ceftiofur is the length of time that concentrations exceed the MIC [47]. It would be important to know tissue concentrations of an antibiotic when considering treatment of *Borrelia* infections and if those concentrations are above the MIC. In the only publication reporting tissue concentrations of ceftiofur following IM administration of ceftiofur crystalline free acid, tissue levels of its metabolite in the uterus were maintained between 0.1–0.2 μg/g, which is above the MIC of *Borrelia* found in our in vitro report [44]. Even more important would be to know the in vivo response to treatment and in the only experimental equine *B. burgdorferi* antibiotic study performed to date, the aqueous solution of sodium ceftiofur at 2.2 mg/kg IM daily for 28 days eliminated *B. burgdorferi* from two of four experimentally infected ponies [48]. Although serum trough levels of sodium ceftiofur, administered at this dose and interval would be predicted to have remained above the MIC for *B. burgdorferi*, trough levels in tissue would have likely been near or below the MIC and this could be one possible explanation why two of the four ponies remained infected [48, 49]. A similar long-acting antibiotic, cefovecin, has recently been shown to decrease the number of dogs with joint lesions and to induce a marked reduction in serum antibody in an in vivo experimental *B. burgdorferi* study [50].

Minocycline and doxycycline have been shown in numerous studies to have similar MICs to *B. burgdorferi* with most MIC results ranging between 0.12–0.63 μg/ml, which are similar to our findings [22]. Minocycline and doxycycline are known to be highly efficacious in treating early onset Lyme disease in humans [51–53]. Bioavailability of the two drugs after per os (PO) administration is significantly lower in horses than humans (20–30% versus 95–100%) and duration of infection prior to beginning treatment is likely longer in horses, therefore, difference in efficacy in treating *B. burgdorferi* between the two species might be expected [33–35, 54]. In the horse, minocycline has better bioavailability than doxycycline, and at the currently recommended dosage of 4 mg/kg PO every 12 h(q12h) provides a peak serum concentration of approximately 0.67 μg/ml which is below the *B. burgdorferi* MIC (0.8μg/ml) found in this study but higher than the MIC found in several other reports [22, 34]. Highest reported trough synovial fluid concentrations of minocycline in horses were 0.33 μg/ml, below the MIC in the current study [34]. Minocycline concentration in the CSF of normal horses dosed at 4 mg/kg q 12 h was 0.39 μg/ml and in aqueous fluid was 0.11 μg/ml ± 0.04 in needle-disrupted blood aqueous barrier suggesting minocycline may have marginal efficacy for treating neuroborreliosis and low efficacy for treating Lyme uveitis [34]. Doxycycline administered at 10 mg/kg is reported to result in a peak serum concentration between 0.32–0.97 μg/ml with a lower percentage distribution into CSF and aqueous fluid than minocycline [33, 55]. In the only experimental equine *B. burgdorferi* antibiotic study, doxycycline administered at 10 mg/kg PO q24h for 28 days was able to eliminate *B. burgdorferi* in only one of four treated ponies [48]. It should be pointed out that the dose of doxycycline used in that 2005 study was less than the current commonly used dose of 10 mg/kg q 12 h. These pharmacokinetic studies and the current and previously reported MIC data may help explain why horses treated, even long term, for *B. burgdorferi* infection with either minocycline or doxycycline often have only modest or no decrease in *B. burgdorferi* serologic titers [18]. There does appear to be some accumulation of doxycycline in synovial fluid due to delayed elimination in horses and both minocycline and doxycycline are commonly reported to reduce stiffness and lameness in field cases of Lyme disease, but this might be a result of their known anti-inflammatory effects on synovium and cartilage [56–58]. In the pony experimental infection and treatment study, oxytetracycline (5 mg/kg administered once daily IV) was the only drug used that eliminated *B. burgdorferi* from all treated ponies and based upon the pharmacokinetic study by Teske 1973, trough levels at that dose would have remained above the MIC_−_ for *B. burgdorferi* [48, 59].

Published studies evaluating in vitro sensitivity of *B. burgdorferi* to metronidazole had significant differences; Sapi (2011) reported an MIC of 0.3 μg/ml, while Brorson (1999) found motile *Borrelia* to be minimally affected by metronidazole even at very high concentrations (516 μg/ml) [30, 31]. Reported differences in in vitro *B. burgdorferi* sensitivity to metronidazole and other antimicrobials may be due to differing strains, methods of broth dilution, incubation periods, and end-point spirochete inoculation concentrations [22, 41]. For instance, our study measured inhibitory concentration as opposed to minimum bactericidal concentration (MBC) effects, which determines the killing of all organisms in the test inoculum. MBC values for *Borrelia* are generally three or more times greater than MIC for tetracyclines, cephalosporins, and metronidazole [22, 28]. In vitro test result differences against different morphologic forms (motile spirochetes or round bodies) of *B. burgdorferi* have also been reported. Sapi (2011) showed *B. burgdorferi* can develop increasing antibiotic tolerance as morphology changes from typical spirochetal form in log phase growth to variant round body “cyst” forms in a stationary phase [30]. Much of the controversy that surrounds Lyme disease in humans pertains to chronic Lyme disease and concern by some that the round morphologic variants of *B. burgdorferi* may play a role in the pathogenesis of chronic Lyme disease [32]. These antibiotic resistant cyst forms are known to occur in vitro more frequently under antibiotic pressure from drugs commonly used to treat Lyme disease [30]. Metronidazole is one of the few antimicrobials that has been shown in one study to be moderately effective in vitro against the round form and at concentrations practically achievable in the patient [30]. The importance of the cyst forms and, therefore, the value of metronidazole treatment is questionable as a recent systematic review of the literature suggested there is no clinical literature to justify specific treatment of *B. burgdorferi* morphologic variants [60]. We know that some equine practitioners in the U.S. are using metronidazole as a treatment for *Borrelia* infections and we were therefore interested in testing the MIC of metronidazole against the free-living spirochete [30, 32]. The in vitro finding of the current study would at least suggest that ceftiofur and minocycline would be preferred over metronidazole for inhibition of the motile *B. burgdorferi* with ceftiofur having the lowest MIC of the three drugs tested.

One important limitation of this study is that measurements of MIC made in vitro cannot be directly applied to in vivo situations. In addition to in vitro testing, differences in pharmacokinetics and dynamics of each drug must be considered along with the immune responses of the patient. Regardless, in vitro testing provides a guide to antimicrobial selection of an antimicrobial with good sensitivity pattern against *Borrelia*. A second limitation to the study was that MIC and not MBC was determined in this study and higher concentration of the drugs would likely be needed to kill the bacteria [42]. Another potential limitation of the study is that the main metabolite of ceftiofur, desfuroylceftiofur, was not available and could not be used in the in vitro study. Although ceftiofur and desfuroylceftiofur, have been shown to have nearly identical activity against all previously tested gram negative bacteria, there is no proof the same would occur with *Borrelia* [43]. Lastly, although we only tested *B. burgdorferi* sensu stricto and not the other genospecies of *B. burgdorferi* sensu *lato*, *B. afzelii* and *B. garinii* which are common in Europe, a previous publication determined that differences in antibiotic (amoxicillin, ceftriaxone, and doxycycline) sensitivity between the three genospecies did not seem sufficiently pronounced to be of fundamental clinical relevance [42].

In summary, this study provides information that might help guide equine practitioners’ decisions on antibiotic treatment of suspected Lyme disease. Based upon current dosing recommendations and pharmacokinetic studies in horses, ceftiofur sodium administered as crystalline-free acid could maintain serum and some tissue drug metabolite concentrations in horses above the ceftiofur MIC for *B. burgdorferi*_._ It is currently unknown if adequate ceftiofur concentrations might be found in tissues most commonly infected by *B. burgdorferi* and if the treatment would actually eliminate the organism. Minocycline, as currently used in equine practice, could maintain serum concentrations near the MIC for *B. burgdorferi* but might not be expected to consistently provide adequate concentration in synovial fluid, aqueous humor or CSF. Metronidazole would only attain MIC against motile *B. burgdorferi* at peak serum concentrations following standard equine dosing. In-vivo treatment studies using either field cases or experimentally infected horses will be required to investigate the efficacy of the antibiotics in treating equine *B. burgdorferi* infections.

## Conclusions

The results of this study provide information to assist practitioners in the therapeutic decision process for treatment of *B. burgdorferi* in horses. Minocycline might provide serum concentrations near or above the MIC for *B. burgdorferi* but may not provide adequate concentration in synovial fluid or CSF. Based upon current dosing recommendations, ceftiofur crystalline-free acid could maintain serum and some tissue concentrations in horses above the MIC for *B. burgdorferi*. Further in-vivo studies will be required to fully elucidate the efficacy of these and other antibiotics in treating equine Lyme borreliosis.

## Abbreviations

AUC: Area under the curve
CSF: Cerebrospinal fluid
IM: Intramuscular
IV: Intravenous
MIC: Minimum inhibitory concentrations
PO: Per os
q12h: Every 12 h
